# Engineered in vivo and in vitro tumor model recapitulates vasculogenic mimicry signatures in melanoma

**DOI:** 10.1002/btm2.10648

**Published:** 2024-01-27

**Authors:** Qizhi Shuai, Xinrui Xu, Yuxiang Liang, Zulala Halbiyat, Xin Lu, Zixuan Hu, Zhiwei Peng, Jie An, Zhiwei Feng, Tingjuan Huang, Hong Zhao, Zhizhen Liu, Jun Xu, Jun Xie

**Affiliations:** ^1^ Department of Biochemistry and Molecular Biology, Shanxi Key Laboratory of Birth Defect and Cell Regeneration, MOE Key Laboratory of Coal Environmental Pathogenicity and Prevention Shanxi Medical University Taiyuan China; ^2^ Laboratory of Ethnopharmacology, Tissue‐Orientated Property of Chinese Medicine Key Laboratory of Sichuan Province West China School of Medicine, West China Hospital, Sichuan University Chengdu China; ^3^ Experimental Animal Center of Shanxi Medical University Shanxi Key Laboratory of Human Disease and Animal Models Taiyuan China; ^4^ Department of Nuclear Medicine The First Hospital of Shanxi Medical University, Collaborative Innovation Center for Molecular Imaging of Precision Medicine, Shanxi Medical University Taiyuan China; ^5^ Department of Hepatopancreatobiliary Surgery The First Hospital of Shanxi Medical University Taiyuan China

**Keywords:** extracellular matrix, melanoma, tumor model, vasculogenic mimicry

## Abstract

Vasculogenic mimicry (VM) describes a process by which tumor cells formed a novel microcirculation pattern in an endothelial cell‐free manner. Clinically, VM is associated with aggressive phenotype and poor patient survival. However, the current models for investigating VM include 2D monolayer cultures, Matrigel‐based cultures, and animal models, each of which has limitations. Matrigel‐based models often exhibit batch‐to‐batch variations, while in vivo tumor models currently produce insufficient amounts of VM. There is currently no suitable tumor model to discover new therapeutic targets against VM. Herein, we establish an extracellular matrix (ECM)‐based engineered tumor model in vivo and in vitro. In this study, we demonstrate that matrix proteins enhanced the VM formation in the engineered xenograft model. Furthermore, we also investigated the role of collagen/fibronectin (FN) in melanoma progression and VM formation. Compared with cells cultured on TCPS plates, the B16F10 cells cultured on collagen/FN coated plates showed increased proliferation and stemness, and significantly enhanced invasion and formation of VM networks. Molecular mechanism analysis showed that Integrin/VE‐cadherin/EphA2/PI3K/MMP‐2 signaling pathways are responsible for VM formation. Our results indicate that collagen/FN matrix plays an important role in VM formation in melanoma, suggesting that ECM protein is a potential therapeutic target for anti‐VM therapy for melanoma.

**Translational Impact Statement**

These in vitro and in vivo engineered tumor models could provide an effective drug screening and evaluation platform for the development of clinical anti‐tumor and anti‐VM drugs, which provide an opportunity for comprehensive understanding of VM‐ECM microenvironment.

## 1 INTRODUCTION

Melanoma is a type of cancer that arises from melanocytes and is widely distributed in the skin and nonskin. Although melanoma accounts for about 5% of all skin cancers, it causes about 75% of skin cancer deaths. Furthermore, the 5‐year survival rate for patients with metastatic melanoma (stage IV) is reduced to 23%.^1^ Angiogenesis inhibitors combined with chemotherapy showed promising efficacy in patients with advanced melanoma in phase 2 clinical trials.^2^, ^3^ However, treatment with VEGF inhibitors alone or in combination with other drugs did not significantly improve overall survival, although patients have been found to have improved progression‐free survival.^4^, ^5^, ^6^

Vasculogenic mimicry (VM) refers to the formation of blood vessels by tumor cells themselves. In the case of uveal melanoma, Maniotis et al. have observed this phenomenon. They found that these tumor cells are capable of remodeling the surrounding matrix to create vessel‐like structures, which in turn facilitates tumor perfusion without the need for angiogenesis.^7^ Clinically, VM patients exhibit poor prognosis and a highly aggressive phenotype.^8^, ^9^ In addition, many reports suggest that VM may play a key role in influencing the overall survival of patients with various types of tumors.^10^, ^11^, ^12^, ^13^, ^14^, ^15^ Many studies have pointed out that some anti‐angiogenic clinical treatments have not been satisfactory possibly due to the presence of VM.^16^ However, the mechanism of VM formation is unclear.

Extracellular matrix (ECM) is a noncellular component that is abnormally expressed in a variety of tumor microenvironments.^17^, ^18^, ^19^ Thus, deciphering the synergy of ECM proteins is key to understanding how the ECM network supports normal vascular function and influences the tumor microenvironment to promote angiogenesis. Collagen fibers are the most predominant ECM component and are strongly remodeled in tumor tissue, which mainly explains the characteristics of the tumor.^20^, ^21^ There are reports that type I collagen is a critical factor to regulate the biological activities of bladder cancer cells. In fact, the collagen‐activated PI3K/AKT signaling pathway enhances the proliferation and colony formation ability of tumor cells.^22^, ^23^, ^24^ A recent study showed that collagen matrix triggers a conserved transcriptional response in tumor cells, leading to the formation of vascular‐like network structures. The feature also correlates with their ability to undergo VM in vitro.^25^ Proteoglycans are heavily glycosylated proteins, major components of the ECM, with various biological functions. Fibronectin (FN) is a multidomain ECM glycoprotein that plays an important role in embryonic development and pathological angiogenesis. In addition to promoting cell adhesion and signaling pathways through receptors, FN matrices also support other matrix protein assembly.^26^, ^27^

Current models for investigating VM include two‐dimensional monolayer culture, Matrigel‐based culture, and animal model. But each approach has its limitations.^28^, ^29^ Traditional 2D cell cultures lack the 3D ECM environment necessary to study tumor cell–microenvironment interactions, and commonly used animal models are relatively simple and have insufficient VM.^30^, ^31^ Additionally, existing Matrigel‐based VM models, which is extracted from the ECM of Engelbreth‐Holm‐Swarm sarcoma, are of great help to the study of VM in vitro,^32^, ^33^ but there are some problems, such as cost intensive, complex components, and batch‐to‐batch difference. These models are not conducive to the development of VM research. Although in vivo models and in vitro cultures of many cell lines have been successful, the need for VM models is particularly urgent.

Toward that end, we establish an ECM‐based engineered tumor model. we select collagen and FN as the main ECM of the tumor microenvironment. Next, we used the tumor cells induced by ECM to improve the differentiation potential of the tumor cells to VM, and further transplanted them into mice. Using the host microenvironment, we promoted the generation of VM in vivo, and constructed an animal model of VM to solve the problem of insufficient existing models. Tumor growth, matrix remodeling, and VM formation in different ECM treatment were assessed using immunohistochemical staining, matrix metalloproteinases analysis, and PAS‐CD31 double staining. We propose a multifunctional in vitro tumor culture model with unique advantage to tune the ECM protein component and simultaneously explore proliferation, colony, invasion, tube formation, and the reciprocal signaling with the cancer cells. Furthermore, our finding of ECM response demonstrated the potential mechanism studies along with VM formation. Thus, these in vitro and in vivo engineered tumor models could provide an effective drug screening and evaluation platform for the development of anti‐tumor and anti‐VM drugs, which provide an opportunity for comprehensive understanding of VM‐ECM microenvironment.

## 2 MATERIALS AND METHODS

### 2.1 Preparation of collagen/FN matrix

To prepare the individual collagen I or FN substrate, the collagen I protein or FN (Solarbio, Beijing, China), was first diluted to 5 μg/mL with phosphate‐buffered saline (PBS) solution and then added to tissue culture‐treated plates (TCPS, Conning, New York, USA). After incubating at 37°C for 2 h, wash with PBS for three times before use. To prepare the collagen/FN substrate, collagen I and FN protein were diluted in PBS to 5 μg/mL and synchronously added into tissue culture‐treated plates (TCPS, Conning, New York, USA). After incubating at 37°C for 2 h, wash with PBS for three times before use.

### 2.2 Cell cultures

B16F10 cell line (mouse melanoma cells) was purchased from Procell Life Science &Technology (Wuhan, China). Cells were stored in 75 cm^2^ culture flasks (Conning, New York, USA) with a density of 2 × 10^6^ cells with a 1640 complete medium (Gibco, California, USA). The cells were cultured in 5% CO_2_ incubator at 37°C. Change the medium every 2 days. Cells were passaged with 0.25% trypsin–EDTA solution (Solarbio, Beijing, China) when cells reached approximately 80% coverage.

### 2.3 Animal experiments

Healthy female ICR mice (5 weeks old, female) were obtained from the Laboratory Animal Center of Shanxi Medical University. All completed live animal experiments were subject to the guidance of the Animal Use Commission (Approval number: SYDL2023018), with the consent of Shanxi Medical University's care regulations and animal experiments). The mice were randomly divided into four groups (*n* = 20): *n* = 5 for PBS group, CO group, FN group, and CF group. In group (a), ICR mice were subcutaneously injected with murine melanoma cells (1 × 10^6^ B16F10 cells in 150 μL PBS) on the right flank. In group (b), B16F10 cells (1 × 10^6^) were then suspended in 150 μL 50 mg/mL collagen solution and injected subcutaneously on the right flank. In group (c), B16F10 cells (1 × 10^6^) were then suspended in 150 μL 50 mg/mL FN solution and injected subcutaneously on the right flank. In group (d), B16F10 cells (1 × 10^6^) were then suspended in 150 μL 50 mg/mL collagen/FN solution and injected subcutaneously on the right flank. On the 12th day, all mice were euthanized and the tumor was excised. Tumor weights were measured and the sections were stained for IHC and CD31/PAS.

### 2.4 Immunohistochemistry (IHC), H&E, and PAS staining

Tumor sections were simply incubated overnight at 4°C with primary antibodies (anti‐Ki67, anti‐Integrin β3, anti‐VE‐cadherin, anti‐Vimentin, and MMP‐2) diluted in PBS containing 10% serum, incubated by secondary antibodies at room temperature for 2 h. The cell nuclei were stained with Mayer's hematoxylin for 3 min. The positive cells of six random fields were counted under 400× magnification. The tumor sections were deparaffinized and dehydrated using a graded series of ethanol solutions and stained with hematoxylin–eosin (H&E). For CD31‐PAS double staining, briefly, the sections were incubated with primary antibodies (anti‐CD31) and secondary antibodies, and oxidized in 0.5% periodic acid for 8 min and in Schiff reagent solution for 20 min. After 10 min of washing with distilled water, the sections were re‐stained in Mayer's hematoxylin for 2 min. The observed images were processed in the same way as IHC staining.

### 2.5 Cell proliferation assay

Cell proliferation assay was performed using the CCK‐8 kit (Solarbio, Beijing, China). In simple terms, B16F10 cells were seeded at a density of 5 × 10^3^ cells per well onto 96‐well TCPS plates coated with collagen I, FN, and collagen I/FN (5 μg/mL). After being cultured at 37°C for 24 h, discard the cell medium and replace it with 100 mL complete medium containing 10% CCK‐8. The cells were incubated at 37°C for another 3 h. The absorbance value for each group of solutions was measured at 450 nm by using a microplate reader (Bio‐Rad, California, USA).

### 2.6 Colony formation analysis

B16F10 cells were seeded at a density of 1 × 10^2^ cells per well onto 6‐well TCPS plates coated by collagen I, FN, and collagen I/FN (5 μg/mL), respectively. After 10 days of incubation at 37°C, the colonies were stained with crystal violet solution (Solarbio, Beijing, China) and then counted. Each experiment was repeated at least three times, independently.

### 2.7 Cell migration assay

Wound healing experiments and trans‐well assays were used to detect the migration capacity of cells. First, a wound healing experiment was performed; B16F10 cells were seeded at a density of 1 × 10^5^ cells per well onto 6‐well TCPS plates coated by collagen I, FN, and collagen I/FN (5 μg/mL). When cell confluency reached 95%, the cells were scraped by using a 200 mL sterile micropipette tip to form a wound. After moistening with PBS solution, mitomycin C (Sigma‐Aldrich, St. Louis, USA) was added to complete medium and incubated at 37°C. Subsequently, the cells were placed under an inverted microscope (Nikon, Tokyo, Japan) at three time points of 0 h, 24 h, and 48 h to observe the healing of the cellular wounds. Three fields of view are randomly selected for each well. Next, a trans‐well (Conning, New York, USA) was performed in which 5 × 10^4^ B16F10 cells were resuspended in collagen I, FN, and collagen I/FN protein solution (5 μg/mL), respectively, then seeded into the upper chamber, and 500 μL medium (10% FBS) was added to the lower well. After being cultured at 37°C for 24 h, the cells were transferred through the trans‐well to the lower chamber, and the cells were fixed by methanol and glacial acetic acid for 15 min, then stained with 10% crystal violet. The chamber was placed under a microscope for pictures. Finally, the migrated cells were calculated.

### 2.8 VM formation and fluorescence labeling

One hundred microliters of ice‐cold Matrigel solution was spread at the bottom of 48‐well plates to allow for even distribution, after incubation in a 37°C incubator for 1 h, then B16F10 cells were digested with trypsin, after which the cells were resuspended in 100 μL different conditioned serum‐free culture media (5 μg/mL collagen I, FN, and collagen I/FN protein solution); 2 × 10^4^ B16F10 cells were inoculated on the Matrigel. Repeat at least three times per group. Incubate at 37°C for 12 h, the wells were photographed using an inverted microscope. Regarding fluorescence labeling, according to the above procedure, VM tubules were stained by Calcein‐AM for 10 min, and the wells were photographed using a fluorescence microscope. Tube numbers were counted.

### 2.9 RT–qPCR analysis

Total RNA from B16F10 cells cultured on the different substrates was obtained by TRIzol reagent (Invitrogen). RT–qPCR assay was performed using Vazyme reagent. After reverse transcription, qPCR was performed using ChamQTM Universal SYBR® qPCR Master Mix (Vazyme, Nanjing, China). The data were analyzed using the CT method and first normalized to β‐actin. The primers of qPCR were listed in Table S1.

### 2.10 Immunofluorescence and cytoskeleton staining

After 24 h of culture in each group and discarded the medium, the cells were fixed with 4% (v/v) paraformaldehyde for 15 min, and then incubated with 1% (w/v) BSA at room temperature for 2 h. After incubation with primary antibody mouse Integrin β3 (1:200, Abmart, China), primary antibody rabbit Vimentin (1:100, CST, USA), or primary antibody rabbit Ki‐67 (1:200, Bioss, China) overnight at 4°C, the cells were washed with PBS, followed by incubation with Dylight 550‐conjugated secondary antibody (Boster, China) at room temperature for 2 h, and washed three times with PBS. Cells were then stained with FITC‐phalloidin (Solarbio, China) for 30 min, washed three times with PBS, and incubated with DAPI (Solarbio, Beijing, China) for 5 min at room temperature. All samples were observed using a confocal laser scanning microscope (Olympus, Japan).

### 2.11 Statistical analysis

Statistical analyses were performed using GraphPad Prism Version 5.0 Windows software (GraphPad, CA). One‐way analysis of variance (ANOVA) was used for data processing and analysis. The number of samples in each experimental group was greater than or equal to three (*n* ≥ 3). All quantitative results were expressed as the mean ± standard deviation, and differences with a *p* value<0.05 were considered statistically significant.

## 3 RESULTS

### 3.1 Establishment and characterization of ECM‐based engineered xenograft model

**FIGURE 1:**
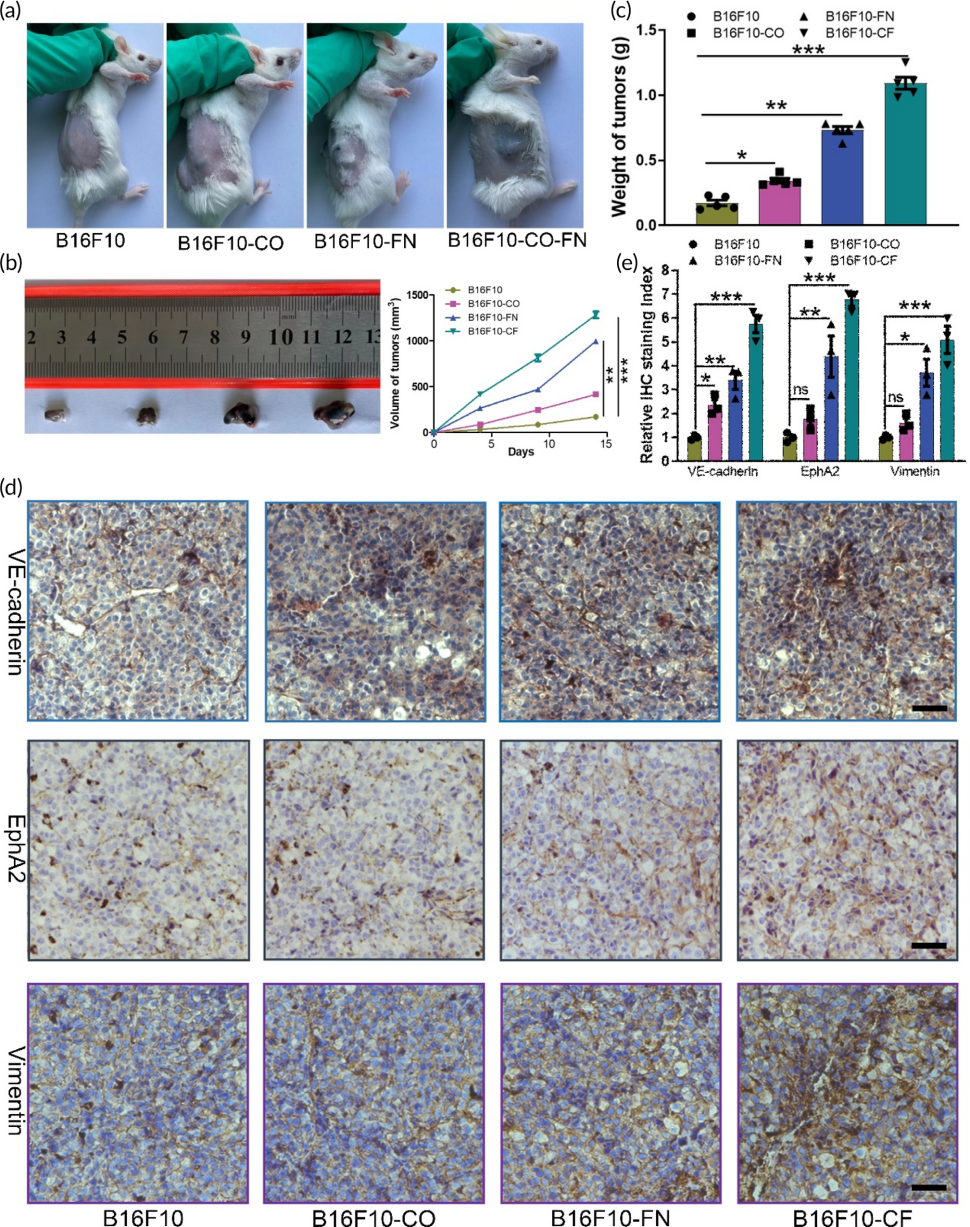
Tumor growth and VM‐related protein expression in an engineered xenograft model. (a) Image of xenograft tumors that were injected with B16F10 cells suspended with PBS, collagen, fibronectin, and CO‐FN protein solutions after 2 weeks. (b) Representative images of tumors from the implanted mice. Tumor volume was recorded every fifth day. (c) Tumor weight was evaluated on the 15th day (*n* = 5 for PBS group, CO group, FN group, and CF group). (d and e) The immunohistochemistry staining, and quantitation of images were to identify the levels of VE‐cadherin, EphA2, and Vimentin staining intensity in the four different groups. Scale bar 50 μm. All data are shown as the mean ± SD, *p < 0.05; **p < 0.01; ***p < 0.001; ns, not significant. VM, vasculogenic mimicry.

To confirm the role of ECM proteins in VM formation of melanoma in vivo, B16F10 cells with or without collagen/FN were subcutaneously implanted into mice to assess the melanoma xenograft tumor progression. Tumor volumes were observed and calculated on day 14 after tumor cells implantation. Collagen/FN proteins promoted melanoma growth in tumor‐bearing mice (Figure 1a). Xenograft tumors collected from mice indicated that collagen/FN proteins notably enhanced tumorigenicity in terms of mean tumor volume (Figure 1b) and weight (Figure 1c). Immunohistochemical staining of Ki‐67 results showed that collagen and FN treatment could better promote melanoma proliferation (Figure S2).

**FIGURE 2:**
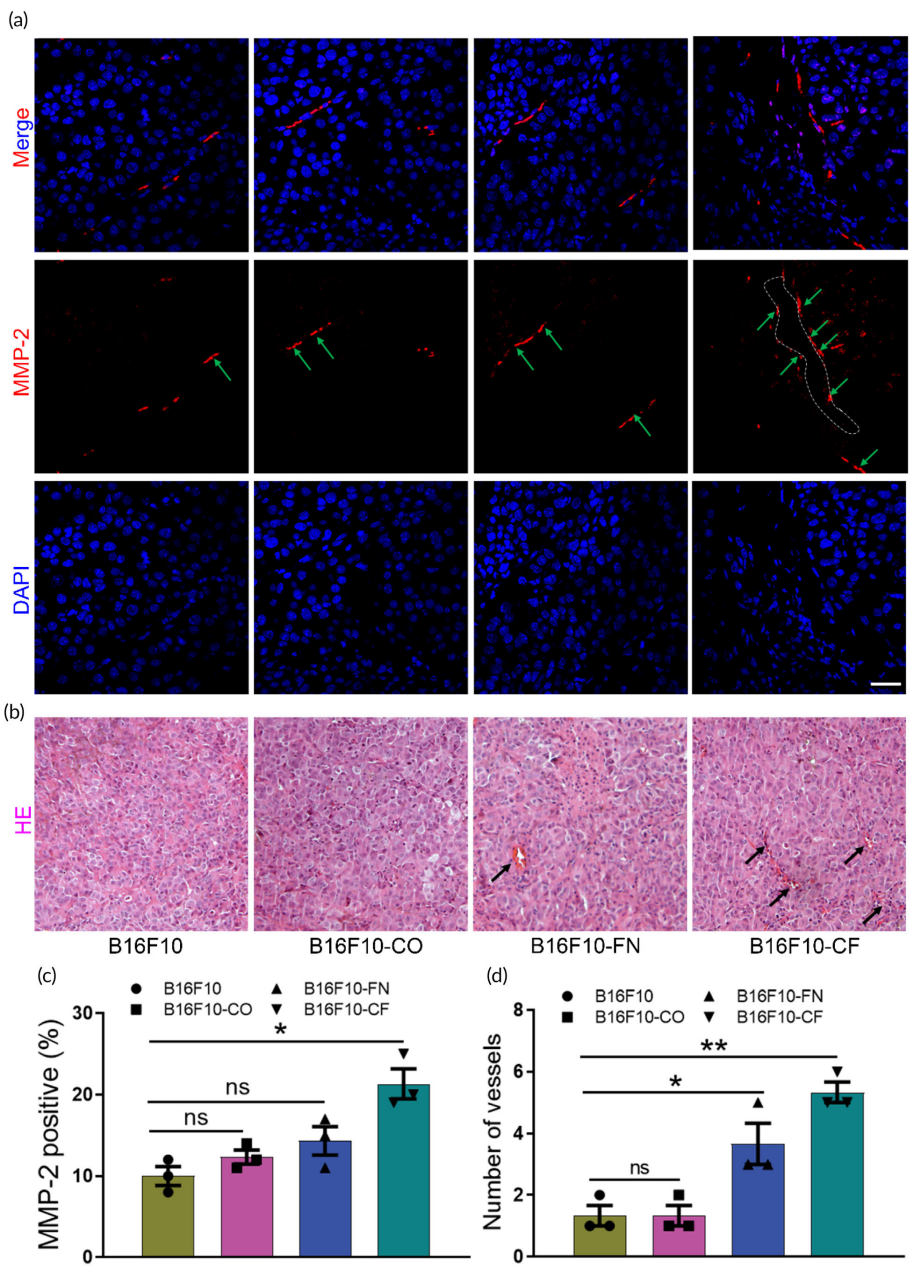
Evaluation of melanoma matrix remodeling and micro‐vessel formation in an engineered xenograft model. (a) The immunohistochemistry staining of MMP‐2 (indicated by green arrows) in the four different groups. Scale bar 30 μm. (b) Analysis of micro‐vessel formation (indicated by black arrows) by hematoxylin and eosin staining in control, collagen, and fibronectin‐treated tumor models. (c) Quantitative analysis of MMP‐2 protein expression levels in the four different groups. (d) Quantitative analysis of vessels in the different xenograft model. All data are shown as the mean ± SD, *p < 0.05; **p < 0.01; ns, not significant.

It is well established that VE‐cadherin plays an important role in the acquisition of VM by aggressive tumor cells.^34^ It is a prominent factor involved in VM, and the down‐regulation of VE‐cadherin expression in melanoma and hepatocellular carcinoma cells inhibits their ability to form VM networks.^35^, ^36^ EphA2 could bind to VE‐cadherin promoter and enhances its activity. It has also been reported that epithelial‐derived tumor cells may differentiate into mesenchymal derived endothelial cells through epithelial‐mesenchymal transition (EMT), which eventually form VM by mimicking the morphology and function of endothelial cells in hepatocellular carcinoma.^37^, ^38^ Therefore, we performed immunohistochemical analyses to investigate the association between VE‐cadherin/EphA2 and EMT in the formation of VM. The immunohistochemical staining results of the xenografted tumor tissues revealed that ECM proteins treatment significantly increased the expression levels of VM and EMT‐related markers, including VE‐cadherin, EphA2, and Vimentin (Figure 1d,e). Furthermore, matrix remodeling is a necessary condition for micro‐vessel formation, and we examined the expression of matrix metalloproteinase‐2 and formation of micro‐vessel in tumor tissues. The immunohistochemical staining of MMP‐2 (Figure 2a,c) and H&E (Figure 2b,d) results of the xenografted tumor tissues showed that collagen and FN treatment could better promote matrix remodeling and micro‐vessel formation.

### 3.2 Collagen and FN synergistically promote VM formation in an engineered xenograft model

**FIGURE 3:**
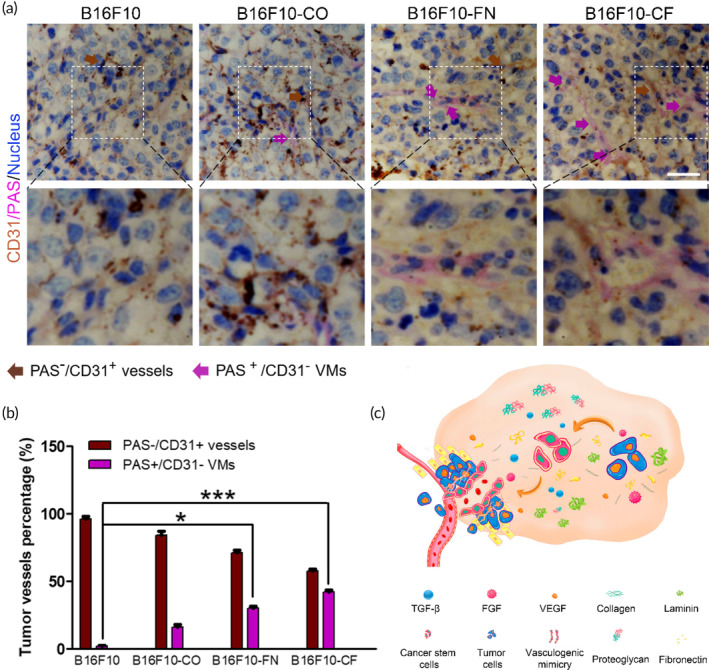
Vasculogenic mimicry and vascularization formation in an engineered xenograft model. (a) PAS/CD31 double staining of xenograft tumors from each group. Brown arrow indicates CD31‐positive blood vessels and pink arrow indicates CD31‐negative/PAS‐positive channels. Scale bar 50 μm. (b) Evaluation of area percentage of VM vessels. The results were presented as mean ± SD, *p < 0.05; ***p < 0.001. (c) Working model illustrating the procession by which ECM promote VM and the vascularization of melanoma in the tumor microenvironment. ECM, extracellular matrix; VM, vasculogenic mimicry.

Unlike normal endothelium vessels, VM is directly surrounded by tumor cells and the basement membrane, which is positive for PAS. CD31 is a marker of endothelial cells, so CD31‐PAS double staining is used to distinguish VM and endothelial‐dependent vessels.^39^ Vessels with positive PAS staining and negative CD31 staining were considered as VM, while vessels with positive CD31 staining and negative PAS staining were considered as endothelial vessels. The CD31‐PAS double staining results of the xenografted tumor tissues showed that ECM proteins treatment significantly increased the number of VM (Figure 3a, b). Hence, these results indicated that collagen/FN could significantly promote melanoma progression and VM formation in an engineered xenograft model.

### 3.3 Collagen and FN matrix enhance the growth and stemness of melanoma

We set out to study the effect of engineered matrix on cellular behavior relevant to tumor progression in vitro, which includes morphological changes, proliferation, colony formation, migration, and invasion. Type I collagen is widely regarded as the main structural protein of the ECM in the tumor microenvironment and plays a significant regulatory role in the tumor progression.^40^, ^41^ In lung cancer, FN expression is abnormal, especially in non‐small cell lung cancer.^42^, ^43^ Moreover, recent evidence suggests that VM formation is frequently observed in ECM‐rich regions containing large amounts of proteins such as collagen and FN.^44^ Consistent with the properties of matrix, alteration in collagen and FN concentration may directly impact cellular behavior. Therefore, we preliminatively investigated the effects of different concentrations of type I collagen and FN on cancer cells (Figure S1). When the concentration of collagen or FN on TCPS was 5 μg/mL, we observed that the proliferation of melanoma cells (B16F10) was markedly increased.

**FIGURE 4:**
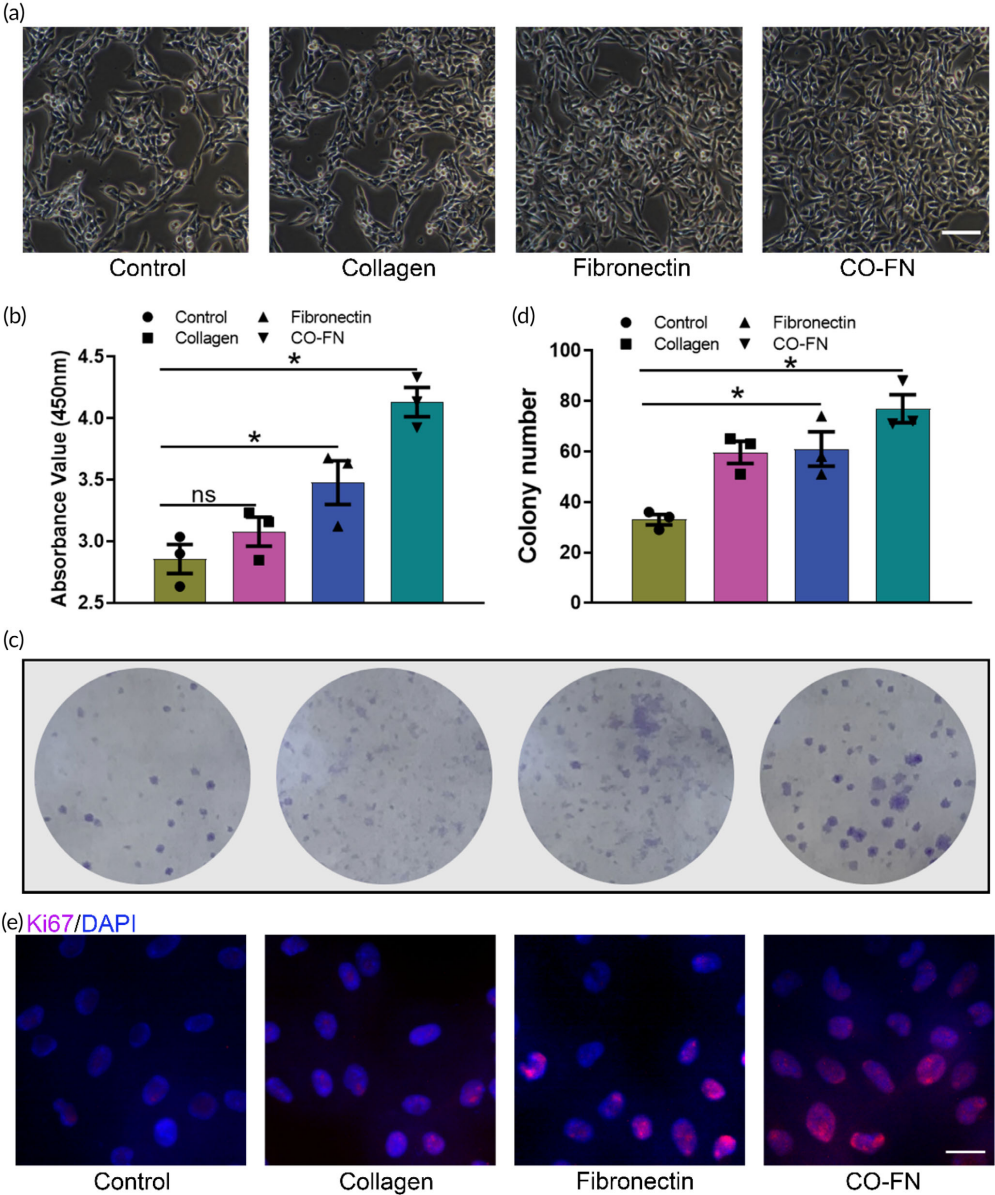
Growth and stemness of B16F10 cells on different matrix. (a) Representative bright field images of B16F10 cells cultured on PBS, collagen (5 μg/mL), fibronectin (5 μg/mL), and CO‐FN coated plates for 24 h. Scale bar 100 μm. (b) CCK‐8 assay of B16F10 cells cultured on PBS, collagen, fibronectin, and CO‐FN coated plates for 24 h. The data was as mean ± SD, *n* = 3. *Significant difference, *p* < 0.05; ns, not significant. (c) Melanoma colony formation was observed on different matrix cultured for 14 days. (d) The quantitative analysis of colony formation images. The data was as mean ± SD, *n* = 3. *Significant difference, *p* < 0.05. (e) Ki‐67 immunofluorescence staining of B16F10 cells cultured on PBS, collagen, fibronectin, and CO‐FN coated plates for 24 h.

Next, the morphologies and proliferation of tumor cells within different matrices were compared with each other by microscopic observation and CCK‐8 assay (Figure 4a, b). While B16F10 showed similar morphologies in all groups, tumor cells in collagen/FN matrix adopted a proliferative phenotype. Similarly, the effect of ECM protein immobilized on the plates on B16F10 cell proliferation was assessed by the CCK‐8 assay. The results showed the proliferation of B16F10 cells in collagen/FN matrix was significantly increased at 24 h compared to TCPS plates (Figure 4b). There is evidence that cancer stem cells in oral squamous cell carcinoma are able to generate VM structures in vivo. Moreover, in melanoma, some phenotypes normally expressed in epithelial or endothelial cells were also expressed in VM‐forming tumor cells.^45^, ^46^ Given the above findings implicating that tumor stemness induces the formation of VM, we sought to determine whether the cells cultured in matrix would increase their stemness. We seeded cells into different culture systems and performed the colony formation analysis. Indeed, the colony formation capability of tumor cells cultured in collagen/FN matrix was strongly increased after 10 days (Figure 4c,d). Moreover, the immunofluorescence staining results showed the number of Ki‐67 positive cells was increased in matrix‐treated cultures (Figure 4e). Collectively, these findings suggested that collagen/FN matrix were able to enhance proliferation and colony formation of B16F10 cells.

### 3.4 Collagen and FN matrix induced cell migration and invasion of melanoma

**FIGURE 5:**
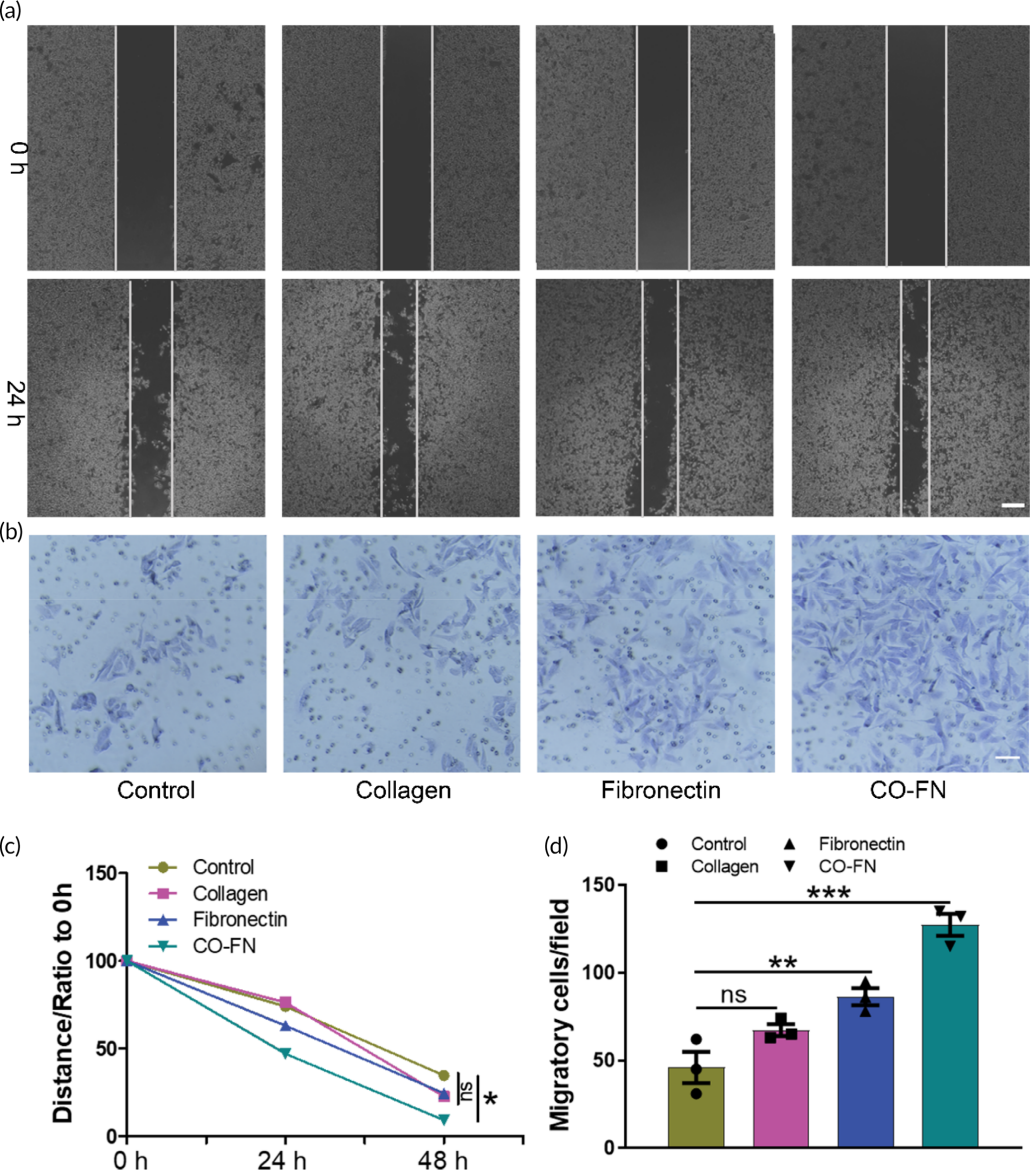
Migration and invasion abilities of B16F10 cells on different matrix. (a) Migration images of B16F10 cells cultured on PBS, collagen, fibronectin, and CO‐FN coated plates for 0 and 24 h. Scale bar 200 μm. (b) Invasion images of B16F10 cells cultured on PBS, collagen, fibronectin, and CO‐FN coated plates for 24 h. Scale bar 100 μm. (c) The quantitative analysis of migration images. (d) The quantitative analysis of invasion images. Randomly select three different fields to be counted in each well. Cell counts per region were averaged from three fields per group (Control, *n* = 3; collagen, *n* = 3; fibronectin, *n* = 3; CO‐FN, *n* = 3). The data were reported as mean ± SD, *n* = 3. ***Significant difference, *p* < 0.001; **significant difference, *p* < 0.01; *significant difference, *p* < 0.05; ns, not significant.

Next, we investigated the influence of matrix on melanoma invasion and metastasis by trans‐well assay. Native type IV collagen has been reported to play a significant role in the invasion process and metastasis of breast cancer.^47^, ^48^ In other words, tumor cells have an increased ability to invade and metastasize on their own before forming VM. Wound healing results showed that the cell migration ability in the CF group was significantly higher than that of cells in the control group (Figure 5a,c). Moreover, the trans‐well assay results showed that the number of B16F10 cells migrating through the trans‐well chamber in the CF group was 3‐fold higher than that in the control group (Figure 5b,d), which was consistent with the result shown in Figure 5c. Therefore, these results indicated that the enhanced migration in B16F10 cells on the matrix could effectively contribute to the formation of VM.

### 3.5 Tube network formation is significantly promoted by matrix

**FIGURE 6:**
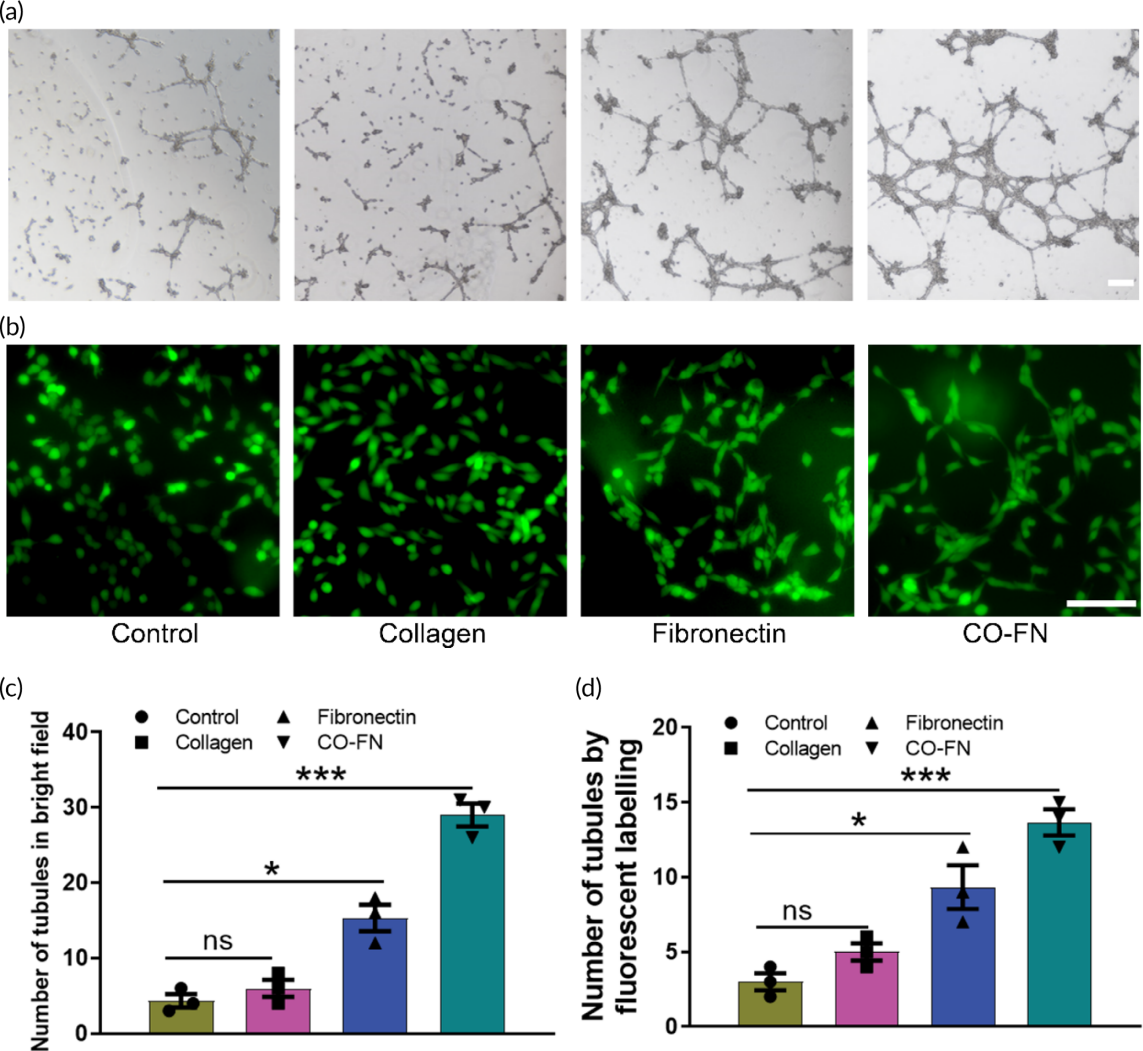
Vasculogenic mimicry formation of melanoma cells in vitro. (a) B16F10 cells were treated with PBS, collagen, fibronectin, and CO‐FN protein for 24 h and tested for VM formation through Matrigel‐based tube formation. Scale bar 200 μm. (b) VM tubules were stained by Calcein‐AM for the wells were photographed using a fluorescence microscope. Scale bar 100 μm. (c and d) Quantitation was shown and results were presented as mean ± SD; tubules counts per region were from three fields per group (Control, *n* = 3; collagen, *n* = 3; fibronectin, *n* = 3; CO‐FN, n = 3), ***Significant difference, *p* < 0.001; *Significant difference, *p* < 0.05; ns, not significant. VM, vasculogenic mimicry.

Tumor growth requires an adequate vascular supply, and VM is increasingly recognized as an important form of angiogenesis. It has been known that ECM may contribute to VM formation, but the synergistic impact of collagen and FN on VM formation remains unclear. We performed a tube formation assay using Matrigel to test the effect of matrix on melanoma VM formation. Surprisingly, we found that melanomas developed more tubes in the presence of matrix proteins (Figure 6a,c). The fluorescent labeling assay also showed similar experimental results (Figure 6b,d). Especially in the CF group, melanoma cells formed a complex VM network. This suggested that matrix proteins can activate VM‐related signaling pathways in melanoma cells, stimulating changes in cell behavioral changes and potentially promoting tumor cell differentiation into VM.

### 3.6 Matrix induces VM characteristics in melanoma through Integrin‐β3/VE‐cadherin/EphA2/PI3K/Vimentin signaling pathway

**FIGURE 7:**
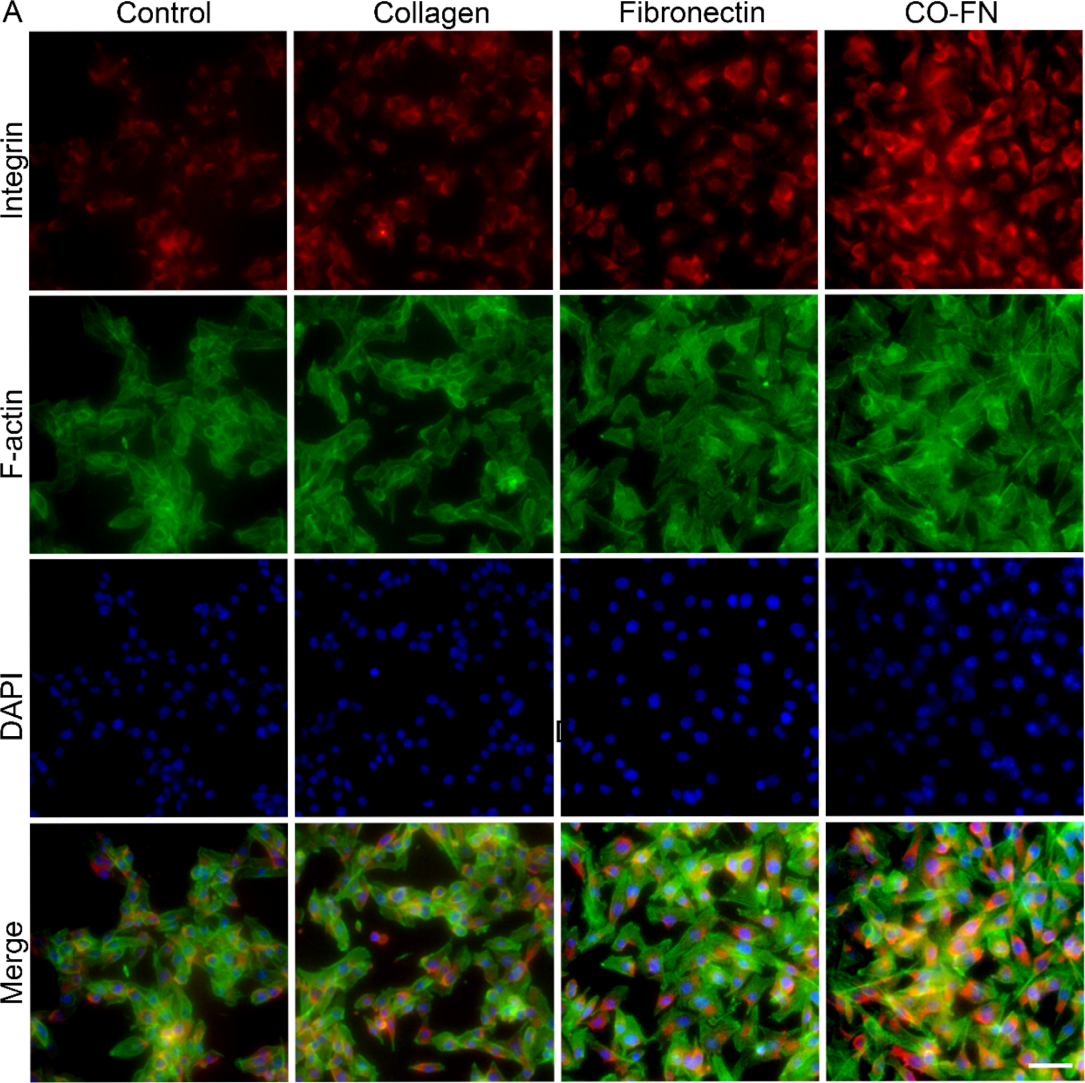
Influence of different matrix on Integrin expression in melanoma. Immunofluorescence staining of Integrin of B16F10 on PBS, collagen, fibronectin, and CO‐FN coated plates for 48 h. Integrin β3 were stained in red by FITC, the F‐Actin were stained by Phalloidin, and the nuclei were stained in blue by DAPI. Scale bar 100 μm.

**FIGURE 8:**
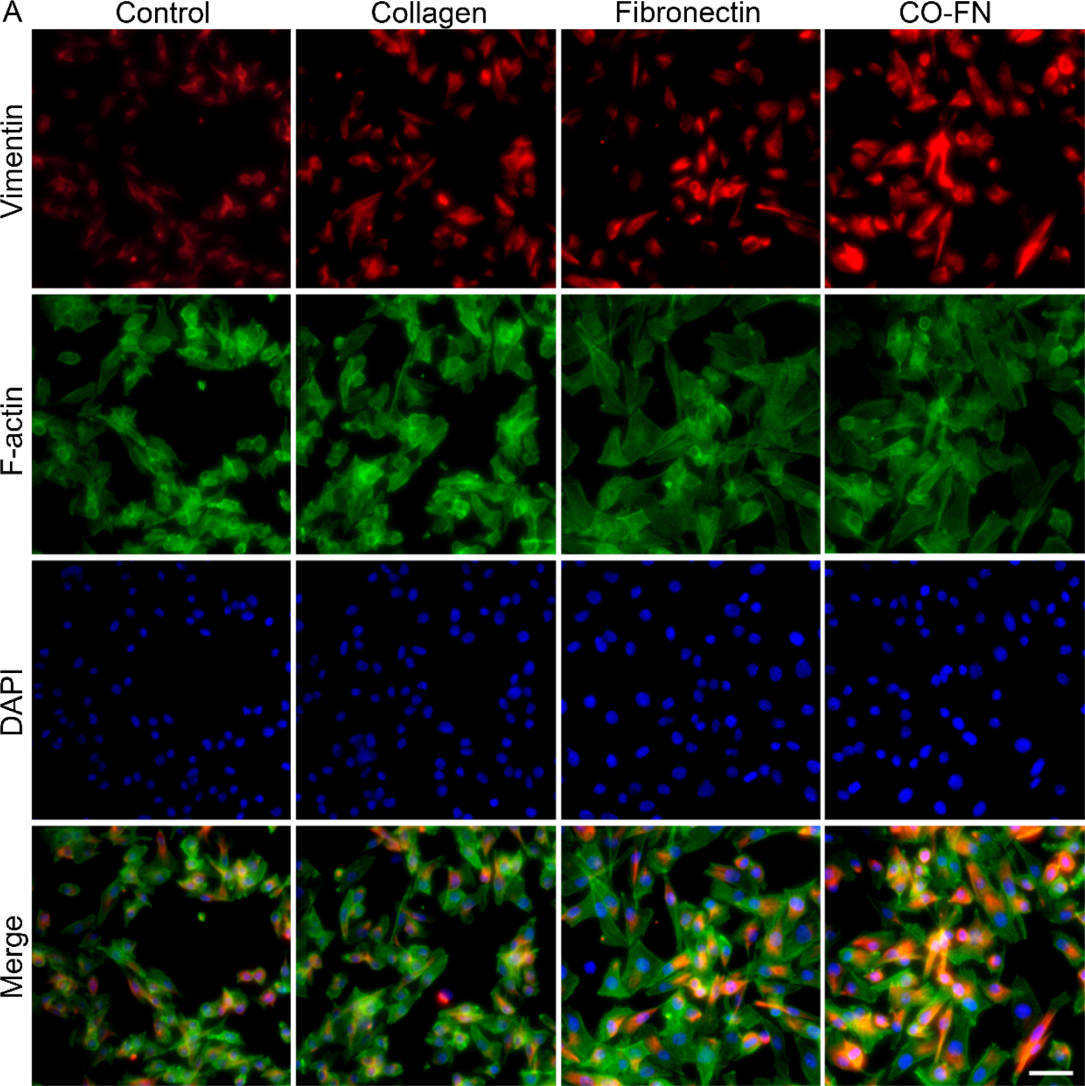
Influence of different matrix on Vimentin expression in melanoma. Immunofluorescence staining of Vimentin of B16F10 on PBS, collagen, fibronectin, and CO‐FN coated plates for 48 h. Vimentin were stained in red by FITC, the F‐actin were stained by Phalloidin, and the nuclei were stained in blue by DAPI. Scale bar 100 μm.

**FIGURE 9:**
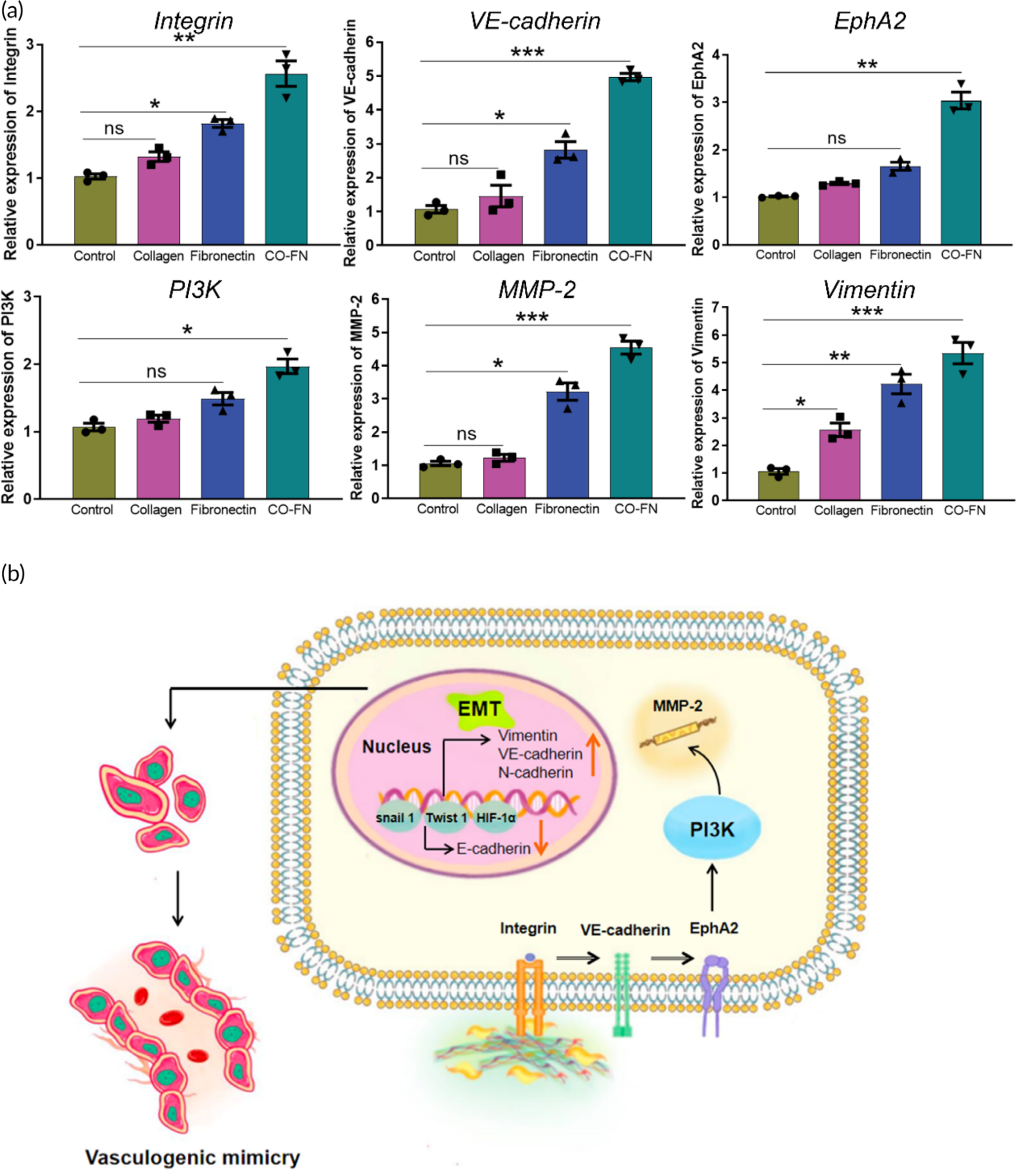
Vasculogenic mimicry signaling pathway in B16F10 cells on different matrix. (a) Gene expressions of Integrin, VE‐cadherin, EphA2, PI3K, and Vimentin of B16F10 cells on PBS, collagen, fibronectin, and CO‐FN coated plates for 48 h were analyzed by qPCR. The data were reported as mean ± SD, *n* = 3. ***Significant difference, *p* < 0.001; **significant difference, *p* < 0.01; *significant difference, *p* < 0.05; ns, not significant. (b) Schematic diagram representing the findings of this study. Collagen and fibronectin matrix stimulate tumor cells by interacting with integrins on the surface of tumor cell membranes. This interaction leads to the presentation of tumor cells to VE‐cadherin and EphA2. As a result, intracellular signal transduction PI3K is promoted, which further enhances the expression of cellular MMP‐2. These processes contribute to matrix remodeling in the tumor microenvironment. Additionally, this stimulation may also promote EMT in tumor cells, causing changes in their behavior and phenotype. Ultimately, this can lead to the formation of VM by tumor cells. ECM proteins may promote VM formation in melanoma via the Integrin/VE‐cadherin/EphA2/PI3K axis and EMT‐related signaling pathways. ECM, extracellular matrix; EMT, epithelial‐mesenchymal transition; VM, vasculogenic mimicry.

Based on the above data and findings that demonstrate matrix participate in formation of VM, but their molecular mechanism is still unclear. Integrins are heterodimeric transmembrane cell surface receptor proteins that mediate binding between cells and the ECM. It has been reported that EGFR could cooperate with integrin αvβ3 to regulate the binding of integrin to extracellular ligands in breast cancer.^49^ To explore signal transduction regulated by matrix, the activation of VM‐related signaling pathways was evaluated by qPCR and immunofluorescence staining. We detected the expression of Integrin and Vimentin by immunofluorescence staining (Figures 7 and 8). In CF groups, the expressions of Integrin and Vimentin of B16F10 cells on matrix were significantly higher than other groups, suggesting that matrix seemed to be able to enhance the Integrin‐mediated intercellular crosstalk of VM in melanoma. The qPCR results showed that the expression of Integrin β3 in B16F10 cells cultured in the CF group was observably higher than that of the cells cultured in the Control group after 48 h culture. Moreover, the expression of VE‐cadherin and EphA2 was dramatically up‐regulated in the CF group compared to the Control group (Figure 9a). Our previous study has demonstrated that VE‐cad‐Fc substrate significantly activated VE‐cadherin/EphA2/PI3K/MMPs in hepatoma carcinoma cells.^50^ EMT is a key process in tumor metastasis. Vimentin is considered to be an important marker in EMT and an important regulator of cell migration.^51^ EMT regulators and EMT‐related transcription factors are highly expressed in VM‐related tumor cells, indicating that EMT may play an important role in VM formation.^52^ In the present study, we also focus on the expression of Vimentin in melanoma. We have shown that Integrin/VE‐cadherin/EphA2/PI3K signaling pathways were responsible for Vimentin expression activated by matrix (Figure 9b).

## 4 DISCUSSION

It is widely recognized that tumors rely on angiogenesis activation to facilitate their growth. Tumors have developed many strategies to trigger what's called “angiogenic switch” to establish connections to the vasculature considered necessary for nutrition and oxygenation.^53^ Tumor vascular formation has a variety of mechanisms, including vessel co‐option, endothelial progenitor cell recruitment, and VM.^54^ VM is a specific blood vessel observed in certain highly invasive tumors in which the blood supply is formed by malignant tumor cells rather than endothelial cells. Many reports have concentrated on the role of angiogenic cytokines in VM.^55^, ^56^ However, there is overwhelming evidence that the matrix is equally significant in ECM remodeling and VM formation.^57^ The ECM can regulate tumor cellular behaviors by functioning as a structural network as well as initiating biochemical signaling pathway in cells through interactions with integrins.^58^ In this engineered tumor model, the cancer cells suspended with ECM solution better simulate the matrix environment by supporting tumor phenotypes, that is, proliferative or migratory, while mimicking the in vivo situation. Our previous studies utilizing VE‐cad‐Fc substrate cultures demonstrated the presence of fusion protein that contribute to the secretion of ECM and cytokine and formation of VM.^50^ Here, we extended these insights to an engineered tumor model with a complex ECM compounds achieved by the incorporation of collagen and FN. In the current study, when cancer cells were cultured in different matrices, collagen/FN matrix regulated tumor cell behaviors such as proliferation, migration, colony, and invasion.

Tumor‐associated stroma is composed of many types of cells, including adipocytes, mesenchymal stem cell, fibroblasts, and immune cells, as well as some ECM proteins, such as FN and tenascin C.^59^, ^60^ In previous reports, increased FN expression in primary tumors plays an important role in poor patient survival.^61^, ^62^ In addition, FN can lead to EMT‐like morphological changes, thereby promoting the migration and proliferation of metastatic cancer cells.^63^, ^64^ However, the mechanistic role of FN in VM formation remains unclear. Collagen, the most abundant ECM component in the stroma, is particularly associated with changes in the stroma during tumorigenesis and is associated with increased stroma density and increased clinical stage of breast cancer.^65^, ^66^, ^67^ Evidence suggested that collagen plays a significant role in the tumor microenvironment changes that accompany tumor progression.^68^, ^69^ A recent study showed that collagen matrix triggers a conserved transcriptional response in tumor cells, leading to the formation of vascular‐like network structures. The feature also correlates with their ability to undergo VM in vitro.^25^ The conventional method to conduct VM in vitro study is Matrigel. However, the phenotypes of different cancer cell lines vary greatly, and the protein component of Matrigel is relatively complex. Our results showed that collagen/FN matrix promoted the formation of VM in vitro. Likewise, the tumor‐bearing model constructed by conventional tumor cell lines is difficult to form VM due to the cell‐intrinsic limitations, which also provides resistance to the development of VM research. In our study, through the induction of ECM proteins, the engineered xenograft model generated more VM structures.

To further investigate the mechanism underlying ECM and VM formation, we proceeded with qPCR analysis and found that collagen/FN may promote VM formation by activating Integrin/VE‐cadherin/EphA2/PI3K axis and inducing EMT‐related Vimentin expression. Therefore, taken together, targeting collagen/FN expression was a potentially useful method to inhibit VM formation in melanoma and decreased anti‐angiogenic therapeutic resistance. The findings suggest that both cell‐intrinsic factors and ECM play a role in the development of VM. Moving forward, our focus will be on identifying inhibitors for VM by targeting the collagen/FN. We will attempt to screen inhibitors that targeted the interaction of the ECM proteins and Integrin, which is a more accurate and effective strategy to inhibit VM. This study effectively developed an ECM‐based VM model in melanoma, accurately recapitulating VM characteristics specific to melanoma. This model holds potential for VM and anti‐cancer drug screening in melanoma. However, further research is required to determine the direct applicability of this system to other tumors, such as liver and breast cancer. We will also consider other tumor microenvironment factors to elaborate on VM formation and their molecular mechanism. In a word, the novelty of this study was that it not only assessed the role of ECM in VM formation but also constructed a new VM tumor model.

## 5 CONCLUSION

In conclusion, we establish an ECM‐based engineered tumor model that generated more VM structures; also, we present a multifunctional in vitro tumor culture model with a characteristic advantage to tune the ECM protein component and simultaneously explore proliferation, colony, invasion, tube formation, and the reciprocal signaling with the tumor cells. Our studies showed that an Integrin/VE‐cadherin/EphA2/PI3K/MMP‐2 axis could potentially regulate VM formation in melanoma. The study demonstrates that the collagen/FN matrix has the ability to enhance VM formation in melanoma. These findings emphasize the adaptability of tumor cells in response to integrin‐mediated cell–ECM interactions. Moreover, this highlights the potential of ECM proteins in constructing in vitro tumor models for studying VM. Thus, these in vitro and in vivo engineered tumor models will provide an effective drug screening and evaluation platform for the development of anti‐tumor and anti‐VM drugs.

## AUTHOR CONTRIBUTIONS

**Qizhi Shuai:** Conceptualization (lead); funding acquisition (lead); methodology (lead); writing – review and editing (lead). **Xinrui Xu:** Conceptualization (lead); methodology (lead); visualization (lead). **Yuxiang Liang:** Funding acquisition (supporting); writing – review and editing (supporting). **Zulala Halbiyat:** Methodology (equal). **Xin Lu:** Methodology (supporting). **Zixuan Hu:** Methodology (supporting). **Zhiwei Peng:** Formal analysis (equal). **Jie An:** Formal analysis (supporting); funding acquisition (supporting). **Zhiwei Feng:** Methodology (supporting). **Tingjuan Huang:** Formal analysis (equal). **Hong Zhao:** Visualization (equal). **Zhizhen Liu:** Project administration (lead); writing – review and editing (supporting). **Jun Xu:** Project administration (lead); supervision (supporting). **Jun Xie:** Conceptualization (supporting); funding acquisition (lead); supervision (lead); writing – review and editing (supporting).

## CONFLICT OF INTEREST STATEMENT

The authors declare no conflicts of interest.

## Supporting information


**Data S1.** Supporting Information.

## Data Availability

The data that support the findings of this study are available from the corresponding author upon reasonable request.
