# Supplementary material for: Engineered in vivo and in vitro tumor model recapitulates vasculogenic mimicry signatures in melanoma
Source: Bioeng Transl Med. 2024 Jan 27;9(4):e10648. doi: 10.1002/btm2.10648 (PMC11256191; doi:10.1002/btm2.10648)
Supplement: Supplementary file 1 — Data S1. Supporting Information. [file BTM2-9-e10648-s001.docx]

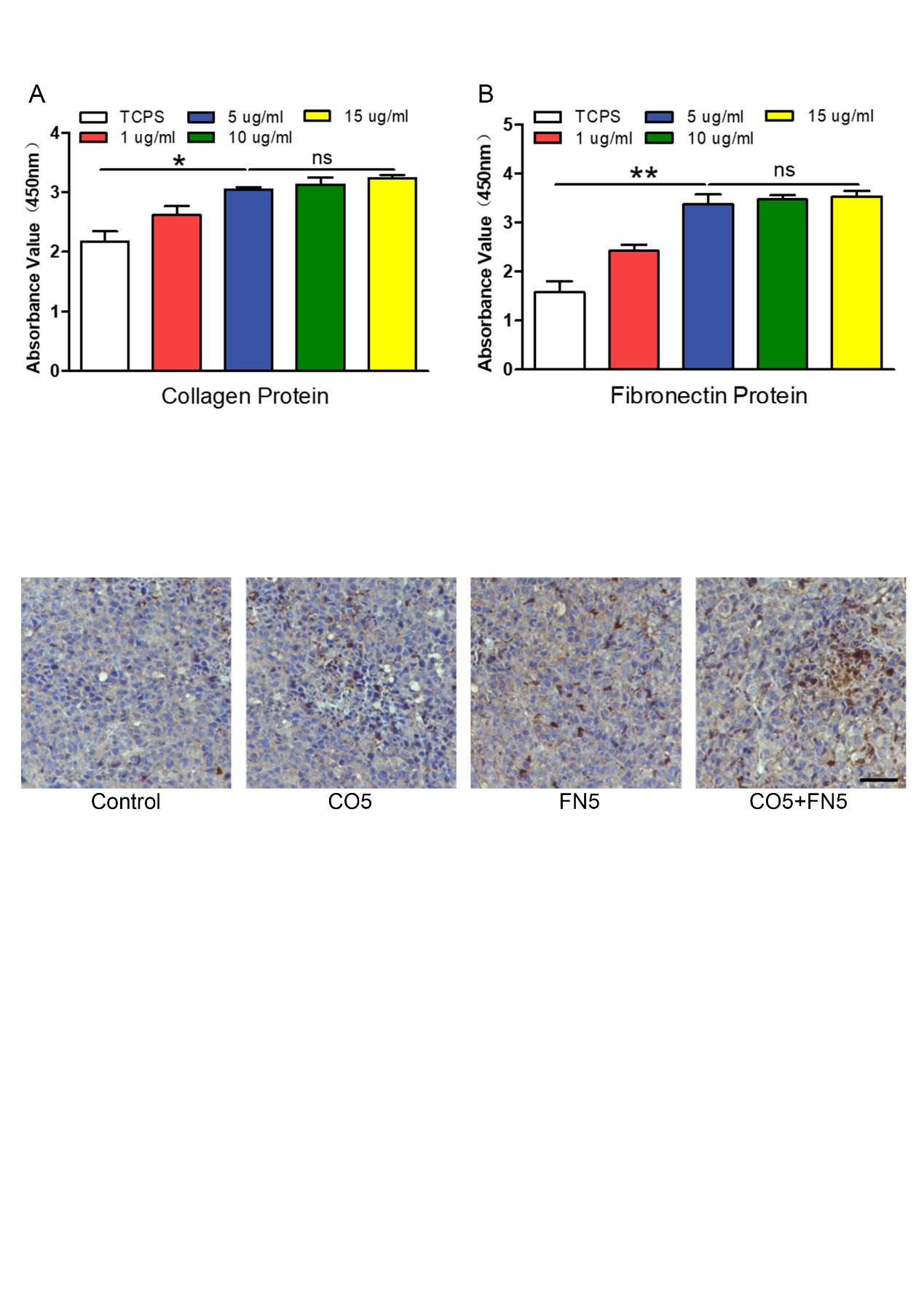


S. 1. Optimization of ECM proteins concentration. (A) The effect of different concentrations of Collagen on B16F10 cell proliferation (0, 1, 5, 10, 15 μg/ml). (B) The effect of different concentrations of Fibronectin on B16F10 cell proliferation (0, 1, 5, 10, 15 μg/ml). The data were reported as mean ± sd, n = 3. *Significant difference, p < 0.05, and ns=not significant.





S. 2. Combining Collagen and Fibronectin treatment to better promote melanoma proliferation using the in vivo mouse model. The immunohistochemistry staining of images was compared to identify the levels of Ki-67 in the 4 different groups (Control, Collagen, Fibronectin and CO/FN). Scale bar 50 μm.

**Table S1:** The primers of qPCR

| Gene | Forward Primer (5'−3') | Reverse Primer (5'−3') |
| --- | --- | --- |
| *VE-cadherin* | CACTGCTTTGGGAGCCTTC | GGGGCAGCGATTCATTTTTCT |
| *Integrin β3* | CCACACGAGGCGTGAACTC | CTTCAGGTTACATCGGGGTGA |
| *EphA2* | GCACAGGGAAAGGAAGTTGTT | CATGTAGATAGGCATGTCGTCC |
| *PI3K* | ACACCACGGTTTGGACTATGG | GGCTACAGTAGTGGGCTTGG |
| *MMP-2* | CAAGTTCCCCGGCGATGTC | TTCTGGTCAAGGTCACCTGTC |
| *Vimentin* | CGTCCACACGCACCTACAG | GGGGGATGAGGAATAGAGGCT |
| *β-actin* | GGCTGTATTCCCCTCCATCG | CCAGTTGGTAACAATGCCATGT |
